# Mortality among Norwegian doctors 1960-2000

**DOI:** 10.1186/1471-2458-11-173

**Published:** 2011-03-22

**Authors:** Olaf G Aasland, Erlend Hem, Tor Haldorsen, Øivind Ekeberg

**Affiliations:** 1 The Research Institute, Norwegian Medical Association, Oslo, Norway; 2 Department of Health Management and Health Economics, Institute of Health and Society, Faculty of Medicine, University of Oslo, Norway; 3 Department of Behavioural Sciences in Medicine, Institute of Basic Medical Sciences, Faculty of Medicine, University of Oslo, Norway; 4 Cancer Registry of Norway, Oslo, Norway; 5 Department of Acute Medicine, Oslo University Hospital, Ullevål, N-0407 Oslo, Norway

## Abstract

**Background:**

To study the mortality pattern of Norwegian doctors, people in human service occupations, other graduates and the general population during the period 1960-2000 by decade, gender and age. The total number of deaths in the study population was 1 583 559.

**Methods:**

Census data from 1960, 1970, 1980 and 1990 relating to education were linked to data on 14 main causes of death from Statistics Norway, followed up for two five-year periods after census, and analyzed as stratified incidence-rate data. Mortality rate ratios were computed as combined Mantel-Haenzel estimates for each sex, adjusting for both age and period when appropriate.

**Results:**

The doctors had a lower mortality rate than the general population for all causes of death except suicide. The mortality rate ratios for other graduates and human service occupations were 0.7-0.8 compared with the general population. However, doctors have a higher mortality than other graduates. The lowest estimates of mortality for doctors were for endocrine, nutritional and metabolic diseases, diseases in the urogenital tract or genitalia, digestive diseases and sudden death, for which the numbers were nearly half of those for the general population. The differences in mortality between doctors and the general population increased during the periods.

**Conclusions:**

Between 1960 and 2000 mortality for doctors converged towards the mortality for other university graduates and for people in human service occupations. However, there was a parallel increase in the gap between these groups and the rest of the population. The slightly higher mortality for doctors compared with mortality for other university graduates may be explained by the higher suicide rate for doctors.

## Background

The relationship between work and health is well documented but complex. In a historical perspective, industrialized western societies have seen a great decrease in work-related diseases, accidents and deaths, mainly due to a reduction in known physical and chemical risk factors, along with improved conditions for employment and regulation of contracts and work hours. Globally, the prevalence of work-related diseases is increasing. Today, developing countries are encountering work-related hazards more frequently than developed countries did at the time of their industrialization, which took place over a relatively long time period [1]. It is estimated that approximately two million work-related deaths occur annually in the world, of which the great majority occur in China, India, and other Asian countries.

Mortality for doctors has been studied for more than a century. In 1886, Ogle found that the mortality rate for the medical profession was "extremely high" [2]. Among other causes, Ogle found that doctors had a higher mortality from liver disease compared with the general population, and he ascribed this to their excessive drinking. It seems that doctors in western industrialized countries had a higher mortality rate than the general population until about 1950 [3,4]. More recently, however, doctors have enjoyed a lower mortality rate than the rate in the general population [4,5], with the notable exception of death from suicide [4,6-11] and violent deaths [4]. Most studies have compared mortality rates for doctors with those of the general population or within the medical profession, but not with comparable socio-economic groups. Some older studies have shown that mortality for doctors is high compared with mortality for other professionals and men in the same socio-economic group [12-15]. However, the results are inconsistent [9]. This may be because the studies are old, the methodology is divergent, the cultural settings are different and the groups are not comparable. In a study from Statistics Norway in which mortality in several occupational groups was compared, covering the period 1970-1980, male doctors (combined with dentists) had the third lowest standardized mortality ratio (SMR) of 0.83, only preceded by teachers (0.80) and farmers (0.73) [16].

The demography and lifestyle of doctors have changed markedly during the last decades, with more female doctors, more informed and demanding patients, economic strains, lower perceived status, and constant attention from the media, patients and health authorities [17,18]. There has also been an increased focus on lifestyle and health. In 1952, 74% of Norwegian doctors smoked daily, whereas the corresponding figure in 1993 was 14% [19]. Thus the prevalence of smoking among doctors has changed from being higher to being much lower than the prevalence in the general population in Norway [20]. Consequently, several studies have found very low mortality rates among doctors from a number of smoking-related disorders [4,6,7]. In Denmark, the standardized mortality ratios for lung cancer and respiratory diseases among doctors compared with the general population for the period 1973-92 were about 0.5 for both genders [4]. Further, specific mortality from heart disease, cancer and diabetes seems to be lower for doctors [21].

Previously, medicine was male dominated. In 1960, 10% of Norwegian doctors were women, compared to 31% in 2000 and 41% in 2008 [22]. Presently, the percentage of female medical students in Norway is 61% [23]. Most previous mortality studies have focused on men. However, when it comes to suicide mortality studies, a consistent finding is a higher rate of suicide in the medical profession, particularly among women [4,10,11]. In a recent meta-analysis, the aggregate suicide rate ratio was 1.4 for male doctors compared to men in the general population, and 2.3 for female doctors compared to women in the general population [11]. A Danish study also found that female doctors have a high mortality rate due to accidents and violent death [4].

It is suggested that if the general population followed doctors on the road to non-smoking, and if the socio-economic gradient between doctors and the general population became less pronounced, then this would have the following effect: The difference in mortality between the general population and doctors would getting less, and the difference in morbidity between the general population and doctors would also get less. However, improvements in mortality and morbidity would occur faster for the general population than for doctors [24].

In a previous study, we investigated the suicide rate for Norwegian doctors compared with the suicide rate for human service occupations and for the general population during the period 1960-2000 [10]. We found that doctors had the highest suicide risk, clearly elevated from other academics. Theologians had a markedly lower risk than other academics. Nurses and police had intermediate or low suicide risk. In the present study we wished to extend the scope to general mortality data. This is the first nationwide study focusing on doctors, other graduates and the general population in a 40-year time period.

We compared doctors with dentists, theologians, other university graduates, nurses, police and the rest of the population. Our hypotheses are 1) that the difference between doctors and other university graduates decreased, but 2) that the mortality gap between doctors and the rest of the population increased.

## Methods

Information on education was taken from four population censuses conducted by Statistics Norway in 1960, 1970, 1980 and 1990, each census year around November 1^st^. In the 1960 and 1970 censuses, education was coded according to information from personal visits to each household. In the 1980 and 1990 censuses, register data for highest education was used to determine education. In the 1960 Census, education was grouped on the basis of an internal list of coding developed by Statistics Norway [25]. From the 1970 Census onwards, education was coded according to the Norwegian Standard Classification of Education, which is compatible with the International Standard Classification of Education [26].

The groups were divided into trained doctors, dentists, nurses, theologians and police; other university graduates (excluding doctors, dentists and theologians); and others (i.e. all other inhabitants in Norway > 20 years), referred to as the "general population" in the following. Theologians and police were included because we wanted to include some non-medical human service occupations. Other human service occupations, such as auxiliary nurse, psychologist and social worker, were not identified as separate groups in the present study because of unreliable or unavailable data for the whole study period.

The groups were differentiated by gender and 5-year age categories above the age of 20. The total number of person-years was 96 709 953, 46 744 079 male and 49 965 874 female. The first five year period was from November 1960 to November 1965, and the last from November 1995 to November 2000.

Statistics Norway provided a file where the time and causes of death were linked to the census data. During the four decades, different versions of the International Classification of Diseases (ICD) were used from ICD-7 to ICD-10. See Table 1 for a detailed list of causes of death.

**Table 1:** ICD codes

		ICD-7	ICD-8	ICD-9	ICD-10
1	All causes	000-999	000-999	000-999	000-999
2	Infectious and parasitic diseases	000-138	000-136	001-139, 2791	A00- B99
3	Cancer	140-207	140-209	140-208	C00-C97
4	Endocrine, nutritional and metabolic diseases	250-289	240-279	240-278, 2792-2799	E00-E96
5	Cardiovascular diseases	330-334, 400-468, 5702	390-458	390-459	I00-I99
6	Sudden death	7952	7824, 795	7981	R960
7	Respiratory diseases	240-241, 244-245, 470-527	460-519	460-519	J00-J99
8	Digestive diseases	530-587	520-577	520-579	K00-K93
9	Diseases in urinary and genital organs	590-637	580-629	580-629	N00-N99
10	Other diseases	Rest. (001-799)	Rest. (001-796)	Rest. (001-799)	Rest. (A00-A98)
11	Accidents	800-866, 900-965	800-845, 880-949	800-848, 870-949	V00-X39, X50-X59, Y85-Y86
12	Poisoning	870-895	850-877	850-869	X40-X49
13	Suicide	970-979	950-959	950-959	X60-X84, Y870
14	Other violent deaths	Rest. (800-999)	Rest. (800-999)	Rest. (800-999)	Rest. (V00-Y99)
15	Unspecified	Blank, 7955	Blank, 7969	Blank, 7995-7999	Blank, R99

### Statistical analyses

The observations were analysed as stratified incidence-rate data in Tables for epidemiologists in the statistical package Stata [27]. Mortality rate ratios were computed as combined Mantel-Haenzel estimates for each sex adjusting for both age and period when appropriate. This method was chosen because of its ability to handle some types of sparse data. A p-value of < 0.05 was considered statistically significant.

## Results

As shown in Table 2, the total overall number of deaths in the study population was 1 583 559. The mortality rate ratios (MRR) for graduates and human service occupations for the whole period were 0.7-0.9 compared with the general population.

**Table 2:** Number of deaths and all-cause mortality rate ratios, 1960-2000

Education	Number of deaths	Men		Women	
		***N***	***Mortality rate ratios (95% CI)***	***N***	***Mortality rate ratios (95% CI)***

Doctors	2 845	2 565	**0.76 (0.73-0.79)**	280	**0.83 (0.74-0.94)**
Dentists	1 413	1 146	**0.75 (0.70-0.79)**	267	**0.72 (0.64-0.81)**
Nurses	12 228	295	**0.87 (0.77-0.97)**	11 933	**0.79 (0.78-0.81)**
Theologians	1 328	1 307	**0.73 (0.69-0.77)**	21	0.71 (0.46-1.09)
Police	1 702	1 691	**0.87 (0.83-0.91)**	11	1.17 (0.65-2.10)
Other graduates	17 981	16 475	**0.71 (0.70-0.72)**	1 506	**0.77 (0.73-0.81)**
Others	1 546 062	810 543	1 (reference)	735 519	1 (reference)

Total	1 583 559	834 022		749 537	

All the male subgroups in both the human service occupations and the other graduates, had significantly lower mortality rates than that for the general population. This was also seen for the women, although the differences were not significant for the female theologians and the police officers due to small numbers.

The male doctors, police and nurses had significantly higher mortality rates than the other graduates group. There were no statistically significant differences between women in human service occupations and other graduates.

Table 3 shows decreasing mortality rate ratios for male doctors during 1960-2000, from 0.90 in 1960-69 to 0.69 in 1990-99. A similar pattern was found for the other human occupations and other graduates group. However, the male theologians and the female nurses had low and stable mortality rate ratios during the whole period. The mortality rate ratio for doctors was not significantly different from the MMRs for other human occupations or other graduates group in the last decade, neither for men nor for women.

**Table 3:** All-cause mortality rate ratios by decade, 1960-2000. For all strata the reference is always the group "Others"

Education	Decade	Men	Women
		***Mortality rate ratios (95% CI)***	***Mortality rate ratios (95% CI)***
		
Doctors	1960-69	**0.90 (0.82-0.99)**	0.81 (0.56-1.16)
	1970-79	**0.79 (0.73-0.86)**	0.87 (0.67-1.13)
	1980-89	**0.75 (0.70-0.80)**	0.98 (0.80-1.21)
	1990-99	**0.69 (0.65-0.74)**	**0.73 (0.60-0.88)**
	
Dentists	1960-69	**0.84 (0.73-0.97)**	0.91 (0.68-1.21)
	1970-79	**0.87 (0.77-0.98)**	**0.57 (0.43-0.76)**
	1980-89	**0.66 (0.59-0.74)**	**0.75 (0.60-0.93)**
	1990-99	**0.70 (0.64-0.78)**	**0.72 (0.58-0.88)**
	
Nurses	1960-69	0.89 (0.69-1.13)	**0.83 (0.79-0.87)**
	1970-79	1.09 (0.84-1.41)	**0.79 (0.76-0.82)**
	1980-89	0.90 (0.72-1.13)	**0.81 (0.78-0.84)**
	1990-99	**0.75 (0.62-0.91)**	**0.77 (0.75-0.80)**
	
Theologians	1960-69	**0.74 (0.65-0.84)**	1.92 (0.72-5.13)
	1970-79	**0.73 (0.66-0.81)**	0.29 (0.04-2.07)
	1980-89	**0.75 (0.68-0.83)**	0.69 (0.31-1.54)
	1990-99	**0.68 (0.61-0.76)**	0.65 (0.35-1.20)
	
Police	1960-69	0.97 (0.84-1.11)	1.66 (0.23-11.81)
	1970-79	0.90 (0.81-1.01)	1.30 (0.33-5.22)
	1980-89	**0.86 (0.79-0.94)**	1.06 (0.34-3.30)
	1990-99	**0.83 (0.76-0.89)**	1.12 (0.46-2.68)
	
Other graduates	1960-69	**0.82 (0.79-0.85)**	**0.73 (0.63-0.84)**
	1970-79	**0.73 (0.71-0.76)**	**0.82 (0.73-0.93)**
	1980-89	**0.71 (0.69-0.73)**	**0.83 (0.76-0.91)**
	1990-99	**0.65 (0.64-0.67)**	**0.72 (0.67-0.78)**
	
Others		1	1

**Figure 1:**
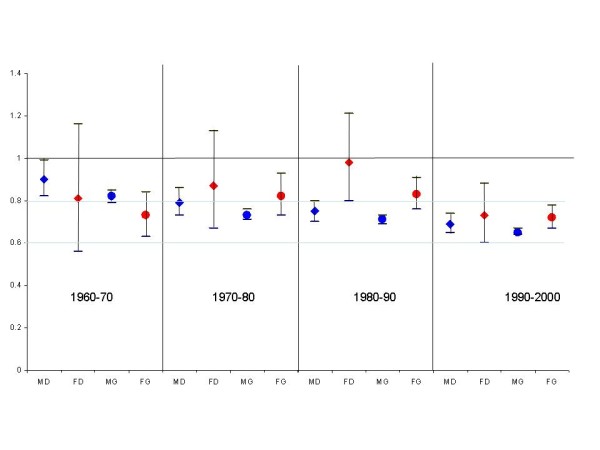
**Mortality relative to the general population (horizontal line) for male and female doctors (blue and red diamonds) and male and female other academic graduates (blue and red circles) in Norway for the four decades between 1960 and 2000**.

Figure 1 compares the mortality rate ratios of female and male doctors with that of other graduates for each of the four decades. There is a clear convergence in these two groups over time, and an increasing gap between the doctors/other graduates and the general population (the horizontal line).

Table 4 shows mortality rate ratios for specific causes of death. Doctors did not differ significantly from other graduates. Compared with the general population, suicide was the only cause of death with a higher MMR for doctors. Other graduates had lower MMRs for all causes of death with the exception of suicide among the females, where we found a slightly elevated MRR (1.31, 95% CI 0.94 - 1.80).

**Table 4:** Mortality rate ratios (with 95% confidence intervals) for some causes of death among doctors and other graduates compared with the general population 1960-2000

Cause of death	No. of deaths	Doctors			Other graduates		
		**N**	**Men**	**Women**	**N**	**Men**	**Women**

All causes	20 826	2 845	**0.76 (0.73-0.79)**	**0.83 (0.74-0.94)**	17 981	**0.71 (0.70-0.72)**	**0.77 (0.73-0.81)**
Infectious and parasitic diseases	177	25	0.80 (0.52-1.22)	1.36 (0.51-3.63)	152	**0.76 (0.65-0.91)**	0.75 (0.43-1.29)
Cancer	5 643	740	**0.87 (0.81-0.94)**	0.85 (0.68-1.06)	4 903	**0.83 (0.80-0.85)**	0.92 (0.85-1.01)
Endocrine, nutritional and metabolic diseases	191	25	**0.51 (0.33-0.78)**	0.73 (0.27-1.95)	166	**0.54 (0.46-0.63)**	**0.41 (0.24-0.70)**
Cardiovascular diseases	9 396	1 258	**0.71 (0.67-0.76)**	**0.71 (0.59-0.86)**	8 138	**0.69 (0.67-0.71)**	**0.65 (0.60-0.71)**
Sudden death	492	59	**0.59 (0.45-0.77)**	1.16 (0.52-2.59)	433	**0.66 (0.60-0.73)**	0.87 (0.60-1.29)
Respiratory diseases	1 453	213	**0.66 (0.57-0.76)**	1.02 (0.71-1.45)	1 240	**0.60 (0.57-0.64)**	**0.76 (0.64-0.91)**
Digestive diseases	528	63	**0.59 (0.45-0.77)**	0.77 (0.38-1.54)	465	**0.68 (0.62-0.75)**	**0.56 (0.40-0.78)**
Diseases in urinary and genital organs	306	30	**0.55 (0.38-0.79)**	0.23 (0.03-1.64)	276	**0.73 (0.65-0.83)**	**0.45 (0.25-0.81)**
Other diseases	1 069	165	0.88 (0.75-1.03)	0.62 (0.38-1.04)	904	**0.67 (0.62-0.72)**	0.85 (0.71-1.02)
Accidents	839	119	**0.74 (0.61-0.90)**	1.36 (0.82-2.26)	720	**0.67 (0.63-0.73)**	0.93 (0.72-1.20)
Poisoning	35	7	0.51 (0.23-1.13)	1.55 (0.22-11.00)	28	**0.29 (0.20-0.43)**	0.72 (0.23-2.22)
Suicide	455	111	**1.77 (1.45-2.16)**	**2.93 (1.70-5.04)**	344	**0.76 (0.68-0.86)**	1.31 (0.95-1.80)
Other violent deaths	25	6	0.72 (0.27-1.92)	2.84 (0.71-11.35)	19	**0.37 (0.22-0.62)**	0.86 (0.32-2.32)
Cause of death not given	217	24	0.70 (0.46-1.05)	0.40 (0.06-2.83)	193	**0.73 (0.63-0.84)**	1.01 (0.62-1.66)

The lowest estimates of physician mortality were for endocrine, nutritional and metabolic diseases, diseases in the urogenital tract or genitalia, digestive diseases and sudden death. These estimates were nearly half of those for the general population.

Moreover, doctors and other graduates had significantly lower MRRs for cancer: 0.87 for male doctors and 0.83 for other male graduates, and 0.85 for women doctors and 0.92 for other female graduates. The corresponding figures for cardiovascular diseases were 0.71 for male doctors and 0.69 for male graduates, and 0.71 for female doctors and 0.65 for female graduates.

In the age group 40-59 years, mortality from cardiovascular diseases among male doctors and other graduates was nearly one half of that in the general population: MRR 0.58 (95% CI 0.50-0.67) for male doctors and 0.57 (95% CI 0.54-0.60) for the other graduates. For female graduates the figure was even lower (0.39; 95% CI 0.28-0.55). Doctors and other graduates showed a pattern of decreasing mortality from cardiovascular diseases during 1960-2000; MMR 0.91 to 0.60 for doctors, and 0.83 to 0.62 for other graduates (Table not shown).

All graduates including doctors had significantly lower mortality from respiratory diseases than the general population. In the age group 40-59 years the MRR for other graduates was 0.39 (95% CI 0.31-0.51) for men and 0.37 (95% CI 0.16-0.82) for women (Data not shown).

## Discussion

The main finding in this study was that doctors, other university graduates and people in human service occupations had lower and decreasing mortality compared with the general population, confirming our second hypotheses. Also, the difference in mortality between doctors and other comparable groups (graduates and human service occupations) decreased over time, confirming our first hypothesis.

Knowledge about how to lead a healthy lifestyle, the possibility to do so, and elimination of risk factors may partly explain this mortality pattern. The fact that other academics have equal or even lower mortality than doctors, may indicate that these groups also have similar knowledge and opportunities. This has been shown to be of special importance in Scandinavia [28].

The increasing differences in mortality between doctors, other academics and the general population is in line with findings from the UK, where trends in mortality showed a relative widening of social differences developing over the period 1970-93[29]. During the period 1960-1999, the average life expectancy at birth for Norwegians increased from 76.0 to 81.1 years for men and 71.0 to 75.5 years for women [30]. It is important for doctors to be healthy not only for themselves, but also for their patients. For example, it has been shown that it is easier for doctors with healthy habits and lifestyles to discuss preventive behaviour with their patients, and they have more credibility [6,31].

The present study shows that doctors had lower mortality rates from lifestyle-related diseases such as cardiovascular diseases, respiratory diseases and metabolic diseases. This is in line with previous findings. Carpenter et al. showed that specific mortality from cardiovascular diseases, lung cancer, other diseases related to smoking, and particularly diabetes, were lower for doctors [21]. Previous studies have also found low mortality from lung cancer [4,7]. In the present study, cancer mortality was significantly lower for doctors.

Although the percentage of daily smokers among doctors has decreased greatly during the last decades, the present study does not show a corresponding reduction in mortality from respiratory diseases. This is probably due to the fact that chronic pulmonary obstructive disease is not singled out as a cause of death in the study, and that the lower prevalence of smoking among doctors was already well established in 1960. Unfortunately we did not have specific data on smoking habits. Rimpelä et al. suggest that in Finland non-smoking cannot explain a low mortality rate from respiratory diseases because Finnish doctors smoke as much as other well-educated groups, but have a lower mortality rate [15]. Thus, they suggest the probability of earlier diagnosis. In western countries, doctors presumably have better access to health services, and the financial resources to obtain good medical care and to practice healthy habits. Some authors state that doctors attend cancer screening more often [31], are more attentive to their health, get diagnosed more often and earlier, and have lower mortality rates. A Norwegian study found that morbidity is lower among doctors, particularly when measured as sickness absence from work [32]. However, many of these statements are not empirically based. Other studies have found that doctors seek help later than other people, and that they therefore have more serious disease when they get professional help [10,33].

Regarding the specific causes of death, the doctors had a higher mortality rate than the general population for only one specific cause of death: suicide. Nearly 5% (13/280) of deaths among female doctors during the period 1960-2000 were due to suicide. These figures are discussed in detail in our previous paper [10].

In this study we did not investigate other causes of death which may be recorded on death certificates instead of suicide. We wanted to look into this because some studies have shown high numbers of accidental poisoning in men, but high suicide mortality from poisoning and injury in women [10,21]. We did not find higher mortality rates from sudden death, accidental death or unknown cause, but on the contrary, lower mortality rates for male doctors and other graduates. This is in contrast to the findings from a Danish study, which showed a relatively high mortality from accidents and other types of violent death for female Danish doctors 1973-92 [4]. One explanation may be different practices for registration of death in different countries [34].

In this study, no specific cause of death for women stood out except suicide. In general, MRRs were quite similar for men and women, indicating that educational level and health behaviour more than gender explain differences in mortality, except for gender-specific diseases.

### Strengths and limitations

The study covers a 40-year time period and it is the longest study period for doctors and other human service occupations published so far. Moreover, the study is nationwide and includes many professional groups. Despite the 40-year time period, the number of deaths for some causes of death is inevitably rather low. Thus, some of the groups are too small for differences to be statistically significant. Examples of small groups are male nurses and professional women, except female nurses.

Although data on whether professionals were practising or not were available, the quality of these data was not sufficient. However, for people in human service professions, and particularly for doctors, there is a high concordance between education and profession practiced.

Some previous studies have focused on subgroups of diseases, such as diabetes and lung cancer. However, in the present study we have focused on the major disease groups (Table 1).

## Conclusion

Norwegian doctors have lower mortality rate ratios than the general population. The differences between doctors and the general population increased during the 40-year period, whereas the mortality rate ratios for doctors and other university graduates converged.

## Competing interests

The authors declare that they have no competing interests.

## Authors' contributions

OGA, ØE and EH conceived the study and participated in its design and coordination.

TH participated in the design of the study and performed the statistical analyses.

All authors read and approved the final manuscript.

## Pre-publication history

The pre-publication history for this paper can be accessed here:

http://www.biomedcentral.com/1471-2458/11/173/prepub
